# Biological molecular layer classification of muscle-invasive bladder cancer opens new treatment opportunities

**DOI:** 10.1186/s12885-019-5858-z

**Published:** 2019-06-28

**Authors:** Lucía Trilla-Fuertes, Angelo Gámez-Pozo, Guillermo Prado-Vázquez, Andrea Zapater-Moros, Mariana Díaz-Almirón, Jorge M. Arevalillo, María Ferrer-Gómez, Hilario Navarro, Paloma Maín, Enrique Espinosa, Álvaro Pinto, Juan Ángel Fresno Vara

**Affiliations:** 1 Biomedica Molecular Medicine SL, Madrid, Spain; 2 Molecular Oncology & Pathology Lab, Institute of Medical and Molecular Genetics-INGEMM, Hospital Universitario La Paz-IdiPAZ, Madrid, Spain; 3 Biostatistics Unit, Hospital Universitario La Paz-IdiPAZ, Madrid, Spain; 4 Department of Statistics, Operational Research and Numerical Analysis, Universidad Nacional de Educación a Distancia (UNED), Madrid, Spain; 5 Department of Statistics and Operations Research, Faculty of Mathematics, Complutense University of Madrid, Madrid, Spain; 6 Servicio de Oncología Médica, Hospital Universitario La Paz-IdiPAZ, Madrid, Spain; 7 Biomedical Research Networking Center on Oncology-CIBERONC, ISCIII, Madrid, Spain

**Keywords:** Muscle-invasive bladder cancer, Molecular subtypes, Personalized medicine, Androgen receptor, Immune status

## Abstract

**Background:**

Muscle-invasive bladder tumors are associated with a high risk of relapse and metastasis even after neoadjuvant chemotherapy and radical cystectomy. Therefore, further therapeutic options are needed and molecular characterization of the disease may help to identify new targets. The aim of this study was to characterize muscle-invasive bladder tumors at the molecular level using computational analyses.

**Methods:**

The TCGA cohort of muscle-invasive bladder cancer patients was used to describe these tumors. Probabilistic graphical models, layer analyses based on sparse k-means coupled with Consensus Cluster, and Flux Balance Analysis were applied to characterize muscle-invasive bladder tumors at a functional level.

**Results:**

Luminal and Basal groups were identified, and an immune molecular layer with independent value was also described. Luminal tumors showed decreased activity in the nodes of epidermis development and extracellular matrix, and increased activity in the node of steroid metabolism leading to a higher expression of the androgen receptor. This fact points to the androgen receptor as a therapeutic target in this group. Basal tumors were highly proliferative according to Flux Balance Analysis, which makes these tumors good candidates for neoadjuvant chemotherapy. The Immune-high group showed a higher degree of expression of immune biomarkers, suggesting that this group may benefit from immune therapy.

**Conclusions:**

Our approach, based on layer analyses, established a Luminal group candidate for therapy with androgen receptor inhibitors, a proliferative Basal group which seems to be a good candidate for chemotherapy, and an immune-high group candidate for immunotherapy.

**Electronic supplementary material:**

The online version of this article (10.1186/s12885-019-5858-z) contains supplementary material, which is available to authorized users.

## Background

Bladder cancer was estimated to account for 81,190 new cases and 17,240 deaths in the United States in 2018 [1]. Muscle invasive bladder cancer (MIBC) is characterized by a high risk of relapse and metastasis [2]. The standard treatment consists of neoadjuvant chemotherapy followed by radical cystectomy. Nevertheless, neoadjuvant chemotherapy is a cisplatin-based schedule that is associated with significant toxicity. Some patients do not benefit from this approach, with their tumors progressing despite the administration of chemotherapy. Therefore, these patients are receiving a toxic and unnecessary treatment, as well as delaying a potentially curative treatment, such as surgery. Unfortunately, there are no reliable biomarkers to guide the selection of patients for these therapies. Several translational studies have aimed to identify subgroups of patients with different clinical behavior.

Choi et al. identified three groups of MIBC (luminal, basal and p53-like) with different response to neoadjuvant chemotherapy [3]. The Cancer Genome Atlas (TCGA) developed a molecular classification of MIBC based on RNAseq data and hierarchical cluster analysis [4]. In this work, five different groups were established: luminal-papillary (which included luminal tumors with papillary histology), luminal-infiltrated (characterized by lymphocyte infiltration), luminal, basal/squamous (also with lymphocyte infiltration) and a small neuronal group.

Seiler et al. associated TCGA molecular subtypes with response to neoadjuvant chemotherapy in a new cohort of patients [5]. Basal tumors appeared to benefit most from neoadjuvant chemotherapy, whereas luminal immune infiltrated tumors had a worse prognosis. However, these findings are not compelling enough to drive clinical decisions, so further insight into the molecular biology of MIBC is needed.

Data were analyzed using three mathematical methods that have proven to be very useful in other fields. Probabilistic graphical models (PGM) can identify differences in biological process among tumors [6–10]. Mathematical classification methods, such as sparse k-means [11] and Consensus Cluster [12], previously demonstrated their usefulness in the establishment of tumor subtypes [6]. On the other hand, Flux Balance Analysis (FBA) is a widely used approach for modeling biochemical networks. FBA could be used to calculate tumor growth rate [13].

In this study, data from the TCGA cohort were analyzed through PGM and computational analysis to characterize MIBC at the functional level.

## Methods

### TCGA cohort: data pre-processing

TCGA RNAseq data from patients with MIBC and treated with surgery alone were used to perform this study. Patients treated with neoadjuvant therapy were initially excluded from computational analysis in order to analyze the most homogeneous cohort as possible. For survival analysis (which relied on clinical information), subjects who had received targeted therapies or radiotherapy were excluded, as well as those with M1 disease or missing T-stage information.

Log2 of the data was calculated and, as quality criteria, genes detected in less than 75% of the samples were discarded. Missing values were imputed using a normal distribution with Perseus software [14], as previously described [7].

### Probabilistic graphical models

The 2345 genes with highest variation in expression (standard deviation > 2) were selected to build the PGM. The PGM method is compatible with high-throughput data with correlation as associative coefficient, as previously described [6–9]. Briefly, gene expression data were used without other a priori information and the analyses were done using *grapHD* package [15] and R v3.2.5 [16]. PGMs are undirected acyclic graphs dependent on obtaining the spreading over the tree that maximizes the likelihood and them choosing the graph which preserved the decomposability and minimizing the Bayesian Information Criterion (BIC) with the simplest structure [17]. The resulting network was split into several branches and the most representative function of each branch was established by gene ontology analyses using DAVID 6.8 webtool, as previously described. [18]. “*Homo sapiens*” was used as background and categories Biocarta, GO-FAT and KEGG were selected. Functional node activities were calculated by the mean expression of the genes related to the main function assigned to each node.

### Biological layer analyses

Sparse k-means [11] and CCA [12] were used to explore molecular groups in the TCGA MIBC data, as previously described [6]. Sparse k-means assigns a weight to each variable, based on its relevance in the sample classification. Then, a CCA using variables that were selected by the sparse k-means method was applied to define the optimum number of groups for each case. The sparse k-means-CCA workflow was performed several times to explore the presence of independent informative molecular layers. Once the relevant genes for one molecular layer were identified, these genes were removed from the dataset and the sparse k-means-CCA workflow was performed again, allowing the identification of different layers of molecular information and establishing different classifications based on various molecular features. Gene ontology analyses were performed for each layer to derive functional information. Sparse k-means was performed using *sparcl* package [11] and CCA was performed using *ConsensusClusterPlus* package [12] and R v3.2.5 [16].

### Flux balance analysis and flux activities

FBA was used to build a computational model that predicts tumor growth rates. The COBRA Toolboox available for MATLAB [19], and the human whole metabolic reconstruction Recon2 [20] were used. The biomass reaction, representative of tumor growth, which is included in the Recon2, was designated as the objective function. As described in previous works [8, 9], expression data was included into the model by solving GPR rules and using a modified E-flux algorithm [9, 21]. Briefly, “AND” expressions were solved as the minimum and “OR” expressions were solved as the sum. Once the GPR rules were solved, the values were normalized to an interval [0,1] using the max-min function and introduced them at the reaction bounds using the E-flux algorithm [8, 9, 21].

As in previous works [9], flux activities for each pathway were calculated by the sum of fluxes for all reactions involved in one pathway as defined in the Recon2. Then, comparisons between luminal and basal groups were performed using a Mann-Whitney test.

### Statistical analyses

GraphPad Prism v6 was used for basic statistical analyses. All *p*-values were two-sided and considered statistically significant below 0.05. Network analyses were performed using Cytoscape software [22].

## Results

### Data pre-processing and patient selection

The TCGA cohort included 427 patients. Ten patients who had received neoadjuvant chemotherapy were excluded, leaving 417 participants for subsequent analyses. Patients treated with targeted therapy or radiotherapy; M1 at diagnosis or no specified muscle-invasive type in the database were excluded from the analyses involving clinical data. Therefore, 178 patients were used for survival analyses (Additional file 1: Figure S1).

### Patient and sample characteristics

Data from the 178 patients included in the clinical analyses are summarized in Additional file 1: Table S1. The median overall survival, considering a follow-up period of 5 years, was 42 months and there were 73 death events during this time.

### Functional network

**Fig. 1:**
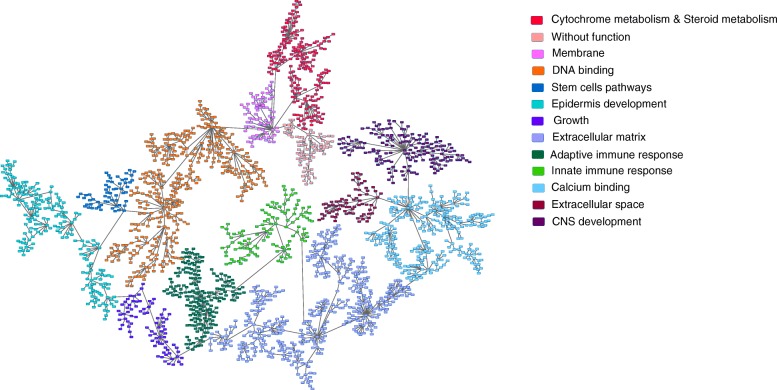
Probabilistic graphical model graph showing the network functional structure. Each functional node is named as its gene ontology main function identified

With the aim of studying differences in biological processes in MIBC, PGM were used to build a network, as previously described [6–9], and the resulting network was functionally characterized. The network included 13 branches or functional nodes, one of them without a main relevant biological function and one with two different main biological functions: cytochrome metabolism and steroid metabolism (Fig. 1). This type of network was useful as a visualization tool to determine differences in biological processes between tumors.

### Biological layer analyses

Biological layer analyses allow us the molecular and immune characterization of tumors [23]. In this case, the sparse k-means-Consensus Cluster Algorithm (CCA) workflow defined 16 different layers of information (Additional file 1: Table S3 and Additional file 2: Table S2). The first three layers had different ontologies and were further analyzed. Layers 4 to 13 had similar ontologies as one of the first three layers, so they were dismissed.

#### Layer 1: extracellular exosome and epidermis development

**Fig. 2:**
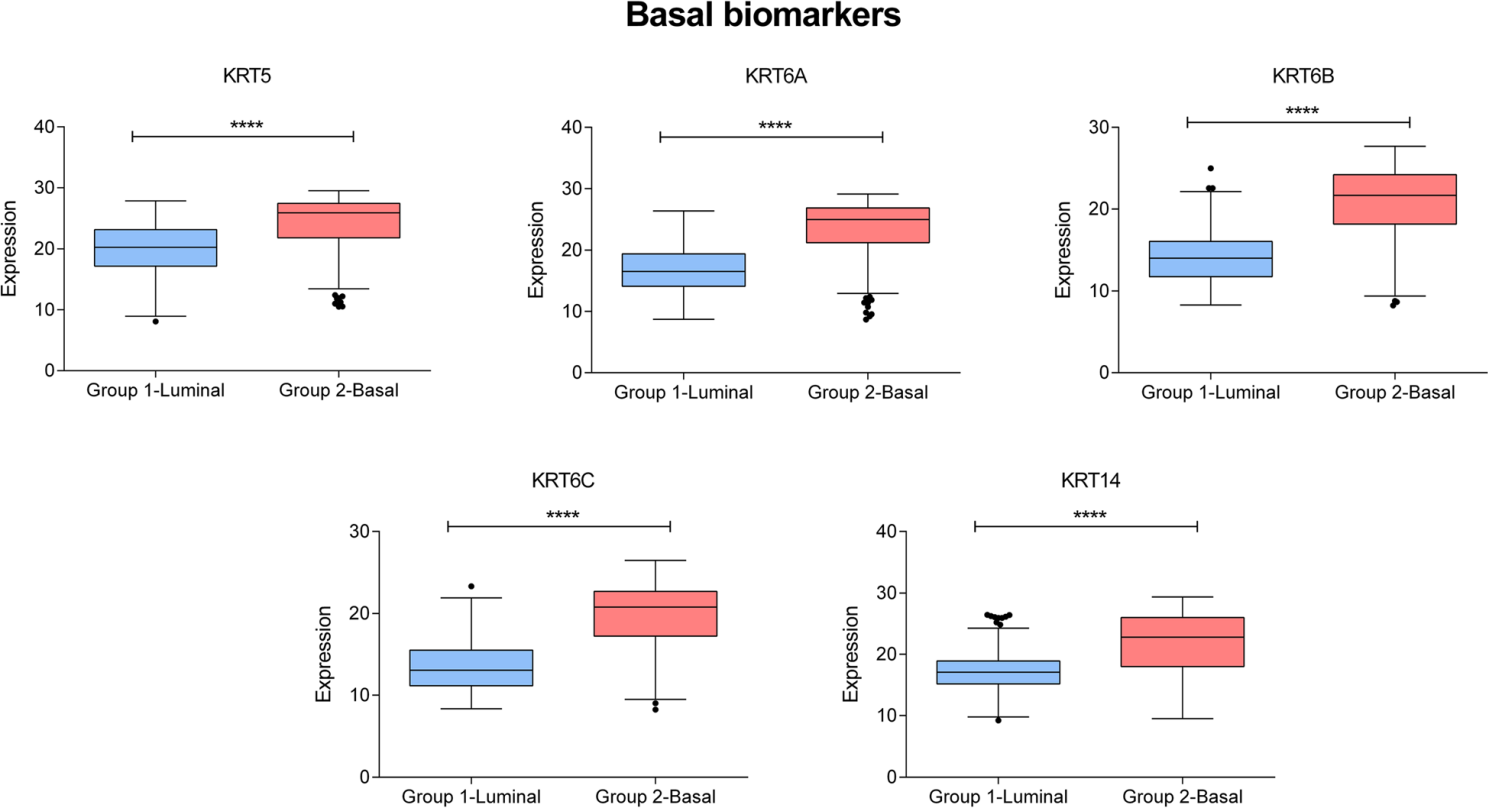
Expression of Basal biomarkers in Group 1.1 and Group 1.2. In the x- axis, Group 1- Luminal: Luminal group defined by layer 1. Group 2- Basal: Basal group defined by layer 1. In the y-axis, gene expression of each biomarker. ****, ≤ 0.0001; ***, ≤ 0.001; **, ≤ 0.01; * ≤ 0.05

**Fig. 3:**
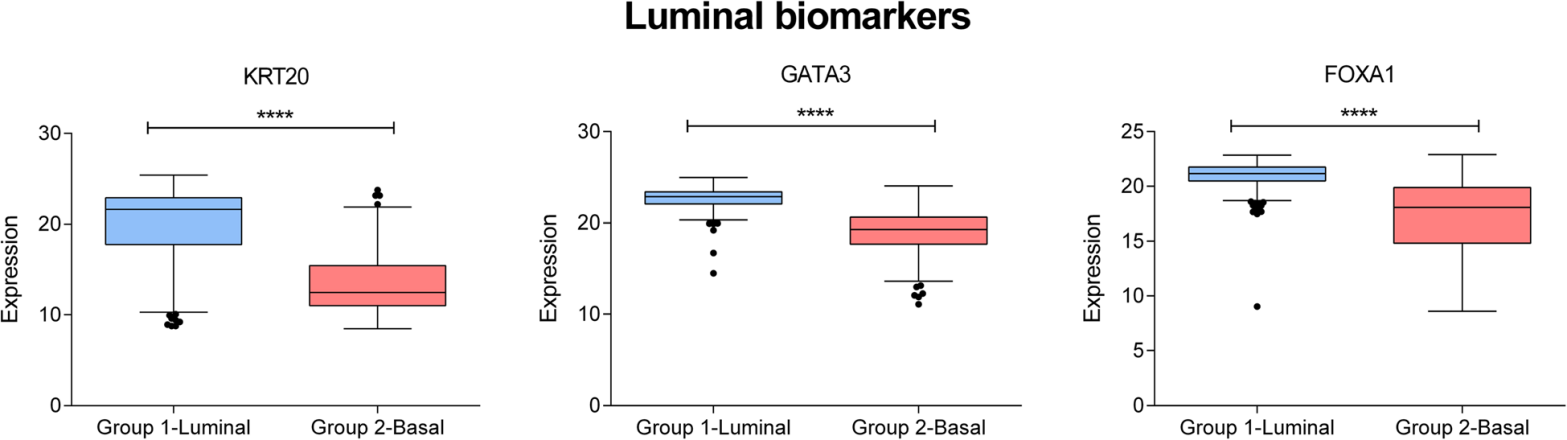
Expression of Luminal biomarkers in Group 1.1 and Group 1.2. In the x-axis, Group 1-Luminal: Luminal group defined by layer 1. Group 2- Basal: Basal group defined by layer 1. In the y-axis, gene expression of each biomarker. ****, ≤ 0.0001; ***, ≤ 0.001; **, ≤ 0.01; * ≤ 0.05

Layer 1 was based on 75 genes, which were mainly related to the extracellular exosome, epidermis development and sodium ion homeostasis. This layer divided patients into two different groups. Group 1.1 included 260 patients (62.35%) and its expression in the network was characterized by a lower expression of genes included in epidermis development and extracellular matrix nodes. Group 1.2 included 157 patients (37.64%) and showed a higher expression of genes included in epidermis development and extracellular matrix nodes (Additional file 1: Figure S2). The TCGA classification of MIBC establishes the existence of both luminal and basal groups. Interestingly, our Group 1.1 had a higher expression of KRT20, GATA3 and FOXA1 genes, all of them considered luminal biomarkers, whereas Group 1.2 had a higher expression of KRT5, KRT6 and KRT14 genes, all of them basal biomarkers (Figs. 2 and 3). So, from now on, Group 1.1 will be called the Luminal group and Group 1.2 the Basal group. Luminal tumors showed a trend towards better survival than basal tumors, although the difference was not statistically significant (*p* = 0.1210, HR = 0.6969) (Additional file 1: Figure S3).

Functional node activity for each node was calculated and compared between these two groups as previously described [6–8]. There were significant differences between luminal and basal subgroups in cytochrome metabolism, steroid metabolism, membrane, stem cell pathways, epidermis development, growth, extracellular matrix, adaptive immune response, innate immune response, extracellular space, and central nervous system (CNS) development functional node activity (Additional file 1: Figure S4).

**Fig. 4:**
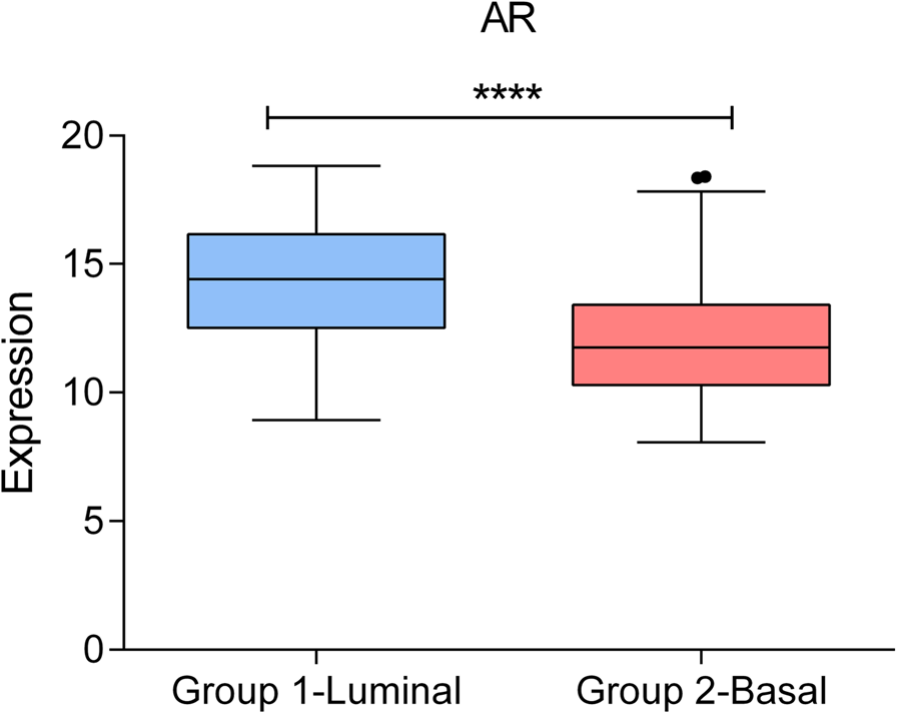
Androgen receptor expression in Luminal and Basal groups. In the x-axis, Group 1-Luminal: Luminal group defined by layer 1. Group 2- Basal: Basal group defined by layer 1. In the y-axis, androgen receptor expression. ****, ≤ 0.0001; ***, ≤ 0.001; **, ≤ 0.01; * ≤ 0.05

Differences in steroid metabolism node between luminal and basal tumors led us to evaluate the androgen receptor (AR) expression in both groups. Interestingly, Luminal tumors showed higher expression of the AR gene (*p* < 0.0001, fold change = 2.669) (Fig. 4).

#### Layer 2: extracellular space

Layer 2 was based on 82 genes mainly related to the extracellular space. Layer 2 classified 268 patients (64.3%) in Group 2.1 and 149 patients (35.7%) in Group 2.2. Group 2.1 was characterized by a higher expression of the extracellular matrix node and lower expression of the cytochrome metabolism node. Group 2.2 had the opposite expression pattern (Additional file 1: Figure S5). Group 2.2 (low extracellular matrix) had a better prognosis than Group 2.1 (high extracellular matrix) (Additional file 1: Figure S6). Therefore, this molecular classification is related to the extracellular matrix process.

#### Layer 3: immune

**Fig. 5:**
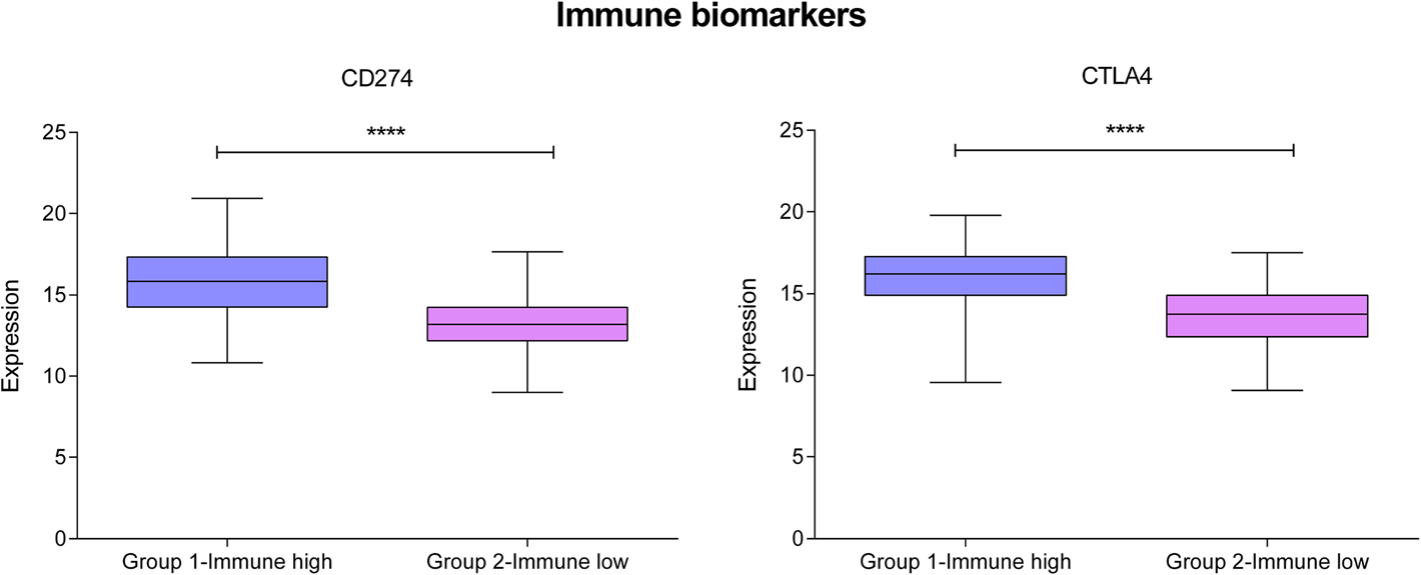
Expression of immune biomarkers in our immune groups. In the x-axis, Group 1-Immune high: Immune-high group defined by layer 3. Group 2- Immune-low: Immune-low group defined by layer 3. In the y-axis, gene expression of each biomarker. ****, ≤ 0.0001; ***, ≤ 0.001; **, ≤ 0.01; * ≤ 0.05

Layer 3 was based on 66 genes mainly related to the inflammatory immune response. This layer divided patients into two groups. Group 3.1 included 215 patients (51.55%) and Group 3.2, 202 patients (48.44%). In the network, Group 3.1 was characterized by a high expression of innate and adaptive immune response nodes so, from now on, it will be called immune-high group. Group 3.2 was characterized by a low expression of innate and adaptive immune response nodes and will be called immune-low group (Additional file 1: Figure S7). The TCGA study used CD274, also known as PD-L1, and CTLA4 to define immune infiltration in both luminal and basal tumors. In our new groups, these two immune biomarkers were more highly expressed in the immune-high group (Fig. 5). In addition, the immune-low group had better prognosis, although the difference was not statistically significant (Additional file 1: Fig. S8). As expected, the node activities of immune nodes were higher in the immune-high group (Additional file 1: Figure S9). Both the basal and the luminal groups contained immune-high and immune-low tumors, although the basal group had less immune-high tumors (Additional file 1: Figure S10).

#### Layers 14 and 15

The first three layers provided distinct ontology information, but layers 4 to 13 contained redundant information because the ontology categories and the resulting classifications were pretty similar. Even though layer 14 (translation) and layer 15 (chemical synapsis) introduced two new ontology categories, they provided no additional grouping information (Additional file 1: Figures S11 and 12).

### Flux balance analysis growth predictions and flux activities

**Fig. 6:**
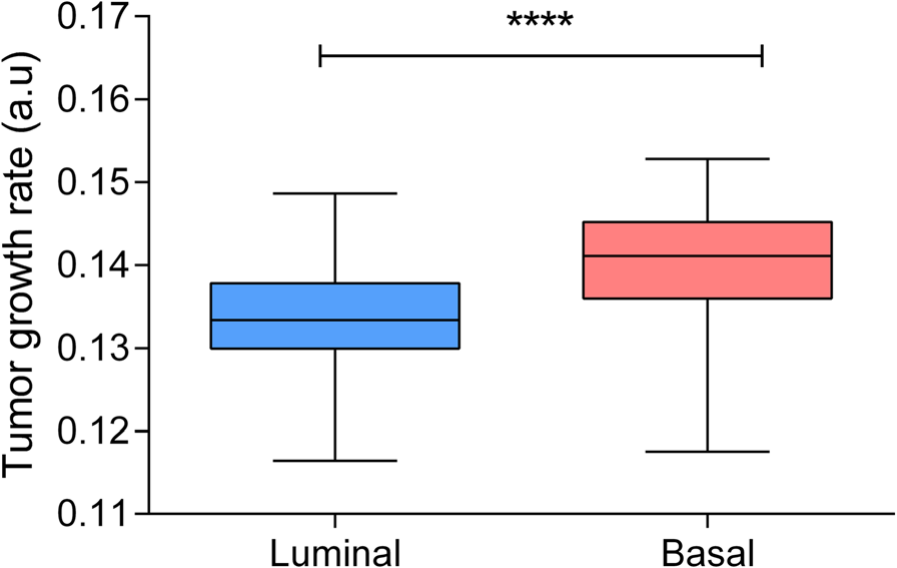
Tumor growth rate predicted by FBA in luminal and basal tumors. ****, ≤ 0.0001; ***, ≤ 0.001; **, ≤ 0.01; * ≤ 0.05 a.u. = arbitrary units

FBA is a computational method widely used to study tumor and microorganism growth [24, 25]. In this study, FBA was used to study tumor growth and compare it between the layer-defined groups. According to FBA predictions, basal tumors were more proliferative than luminal tumors (Fig. 6) (*p* < 0.0001).

Flux activities were calculated for each metabolic pathway and compared between basal and luminal tumors to determine differential metabolic pathways as previously described [9]. Luminal tumors had higher androgen and steroid metabolism flux activity, according to the differences shown in the steroid metabolism and AR expression nodes. Differences were also detected in aminosugar metabolism, CoA synthesis, galactose metabolism, glycolysis, hyaluronic acid metabolism, lysine, methionine, NAD, nucleotide savage, oxidative phosphorylation, phosphatidyl inositol, pyrimidine synthesis, R group, triacylglycerol and vitamin B6 metabolism (Additional file 1: Figure S13).

### Comparison with TCGA classification

The TCGA classification established three luminal groups: luminal, luminal-papillary, and luminal-infiltrated (by lymphocytes), one basal group characterized by an immune positive status, and a small group called neuronal [4]. Our classification established immune information as an independent layer divided between luminal and basal groups, i.e., both of them had immune- high and immune-low tumors. Percentages were similar between both classifications, although we did not identify a neuronal group (Additional file 1: Table S4).

### Relationship between the different layer classifications

Comparing percentages between the different layer classifications (luminal/basal, extracellular matrix-high/extracellular matrix-low, immune-high/immune-low), it was possible to see that the three classifications are complementary to each other (Additional file 1: Table S5). It is true that most of luminal tumors with EM-low features were IM-low, and most of the basal tumors with EM-high characteristics were IM-high. It is remarkable that TCGA luminal-papillary group, which is defined solely by histological features, includes 10 (23% of luminal papillary) tumors with immune-high characteristics when the layer classification was applied. Moreover, we identified a new group formed by luminal, extracellular matrix-high tumors with immune-low features. This group included both papillary (*n* = 4) and non-papillary (*n* = 21) tumors.

## Discussion

MIBC has a poor prognosis, with over 50% relapses in spite of appropriate therapy [26]. Therefore, it is still necessary to characterize MIBC in order to propose new therapeutic targets. Molecular, functional and metabolic characterization by PGM, layer analyses and FBA were performed in this study to provide insight into the molecular features of MIBC.

The PGM unveiled the functional structure of tumors, which has been previously described by our group [6, 8, 9]. This allows for the study of gene expression data from biological and functional points of view. The functional structure obtained by the PGM analyses is a useful visual tool to determine differential biological processes between tumors. As an example of consistency in this regard, cytochrome P450 and UDP-glucuronosyl transferase were related to androgen receptor in the same node, and androgens are known to modulate the expression of these enzymes [27].

Layer analyses provided different information about the molecular features of the tumors, as for example, about the cellular adhesion process and about immune status. This new approach, based on well-known classificatory mathematical algorithms, allows us to study and interpret the molecular information separately from the adhesion and the immune information, which are grouped together within the expression data. The first layer divided MIBC tumors into Luminal and Basal. Dadhania et al. validated Luminal and Basal biomarkers across three different cohorts of patients with MIBC by gene expression and immunohistochemistry [28].

It is remarkable that Luminal tumors presented higher steroid metabolism node activity. Indeed, AR gene presents higher expression in Luminal tumors, suggesting the usefulness of AR as a possible therapeutic target. AR was previously associated with bladder cancer progression [29] and in vitro studies showed that a siRNA against AR decreased proliferation of AR-positive bladder cancer cell lines but had no effect on AR-negative cells [30]. However, the role of AR in bladder cancer remains unclear and further characterization is still necessary. Li et al. showed that more than 30–50% of bladder tumors have a detectable presence of AR [31]. However, they did not relate AR to any molecular subtype. Our proposal is that patients with Luminal MIBC tumors are characterized by a high expression of AR and they could be candidates for therapy with AR inhibitors.

Luminal tumors showed a higher flux activity of androgen and steroid metabolism pathway as well, which agrees with the results found in node activity. Luminal tumors also showed a higher flux activity of the glycolysis pathway so they may respond to drugs targeting metabolism, such as metformin, which has been shown to reduce growth in bladder cancer cells [32].

Moreover, a group of luminal tumors with extracellular matrix-high characteristics was also described. This group is not related to the papillary histology because it had both papillary and non-papillary tumors. As far we know, a group with these features has not been described before.

On the other hand, FBA predicted that, as has been seen in breast cancer [8], basal tumors are more proliferative than luminal tumors. It has been established that basal breast cancer tumors have a good response to chemotherapy because they are more proliferative [33, 34]. Based on the FBA results, the previous knowledge of basal breast tumors, and taking into account that this cohort is chemotherapy-naïve, basal patients may be good responders to chemotherapy as was previously suggested by Seiler et al. [5]. Proliferation has been determined in other tumor types through gene expression, but data in bladder carcinoma are scarce. FBA allows not only to study proliferation but also to compare metabolic pathways.

The third layer identified an immune high expression group with elevated expression of CTLA4 and CD274, which may be a group of patients who are candidates for receiving immunotherapy, given that CD274, also known as PD-L1, and CTLA4 are the basis of current immunotherapy [35]. Interestingly, immune high tumors had a worse prognosis than immune low tumors, consistent with the results reported by Seiler et al., which suggested that luminal immune infiltrated tumors had a worse prognosis [5].

Percentage distribution between luminal and basal tumors was comparable in the TCGA classification and the layer classification. However, the TCGA classification mixes immune, histological and molecular information. Our approach, on the contrary, establishes two independent informative layers: molecular and immune; and, consequently, two complementary classifications. It rendered some interesting findings that complement the TCGA classification: for instance, 10% of basal tumors had an immune-low status, whereas in the TCGA classification all basal tumors had an immune-positive status. Additionally, the TCGA luminal-papillary group, which is defined solely by histological features, had immune-high characteristics when the layer classification was applied. With the arrival of immunotherapies to the clinic, it could be useful to characterize the immune status of tumors and establish groups with differential immune features to drive treatment decisions.

The study has some limitations. Our findings should be validated in an independent cohort. Publications including expression and clinical data are scarce, so validation should be performed in a prospective study. On the other hand, the existence of a neuronal group could not be confirmed. As the neuronal group accounted for a minority of cases in the TCGA study, maybe we should have analyzed a larger population. Finally, although our results suggest that some drugs may work better in specific groups, this should be prospectively validated. Response to chemotherapy, for instance, has been related to multiple factors and the proliferation profile may not be enough to identify responders.

## Conclusions

Computational analyses found different levels of information in gene expression data from MIBC: one of these levels refers to immune features, whereas the other corresponds to the previous classification into luminal/basal subgroups. Our classification may have therapeutic implications for the treatment of MIBC, suggesting that tumors with immune-positive features may be good candidates for receiving immunotherapy, Luminal tumors may be good candidates for androgen receptor inhibitors and Basal tumors may be good responders to chemotherapy.

## Additional files

Additional file 1: **Figure S1**. Flowchart for patient selection. **Figure S2**. PGM’s graph heatmap showing differences between Group 1.1 (Luminal) and Group 1.2 (Basal). Green= underexpressed, Red= overexpressed. **Figure S3**. Kaplan Meier analysis comparing Luminal and Basal MIBC tumors clinical evolution. **Figure S4**. Functional node activities comparison between Luminal and Basal group. **Figure S5**. PGM’s graph heatmap showing differences between Group 2.1 and Group 2.2. Green = underexpressed, Red = overexpressed. **Figure S6**. Kaplan Meier analysis comparing Group 2.1 and Group 2.2 clinical evolution. **Figure S7**. Heatmap showing differences between Group 3.1 (immune-high) and Group 3.2 (immune-low). Green = underexpressed, Red = overexpressed. **Figure S8**. Survival curves obtained for immune groups. **Figure S9**. Immune node activities in immune groups. **Figure S10**. Concordance between classification of layer 1, which divided tumors into Luminal and Basal, and layer 3, which divided tumors into immune-high and immune-low group. **Figure S11**. Heatmap showing differences between groups defined in layers 14 and 15. Green = underexpressed, Red = overexpressed. **Figure S12**. Different classifications obtained by each layer. Colour bars represent the assignation for each patient and the numbers on the righ are the corresponding layers. For instance, layer 12 classified the patients into two groups (light blue and dark blue). **Figure S13**. Flux activities of luminal and basal groups. **Table S1**. Clinical patients’ characteristics. **Table S3**. Main gene ontology term defined for each sixteen layers obtained by the sparse k-means-CCA workflow. **Table S4**. Percentages of patients assigned to each group in TCGA and layer classification. **Table S5**. Number and percentage of tumors assigned to each group by the layer classification. EM = extracellular matrix, IM = immune. (DOCX 2320 kb)

Additional file 2: **Table S2.** Genes that define each layer. (XLSX 43 kb)

## Data Availability

Data sharing is not applicable to this article as no datasets were generated or analyzed during the current study. All data used in this work can be found at https://portal.gdc.cancer.gov/.

## Abbreviations

AR: Androgen receptor
CCA: Consensus Cluster Algorithm
CNS: Central nervous system
FBA: Flux Balance Analysis
MIBC: Muscle-invasive bladder cancer
PGM: Probabilistic graphical models
TCGA: The Cancer Genome Atlas
