# Supplementary material for: Biological molecular layer classification of muscle-invasive bladder cancer opens new treatment opportunities
Source: BMC Cancer. 2019 Jun 28;19:636. doi: 10.1186/s12885-019-5858-z (PMC6599340; doi:10.1186/s12885-019-5858-z)
Supplement: Supplementary file 1 — Figure S1. Flowchart for patient selection. Figure S2. PGM’s graph heatmap showing differences between Group 1.1 (Luminal) and Group 1.2 (Basal). Green= underexpressed, Red= overexpressed. Figure S3. Kaplan Meier analysis comparing Luminal and Basal MIBC tumors clinical evolution. Figure S4. Functional node activities comparison between Luminal and Basal group. Figure S5. PGM’s graph heatmap showing differences between Group 2.1 and Group 2.2. Green = underexpressed, Red = overexpressed. Figure S6. Kaplan Meier analysis comparing Group 2.1 and Group 2.2 clinical evolution. Figure S7. Heatmap showing differences between Group 3.1 (immune-high) and Group 3.2 (immune-low). Green = underexpressed, Red = overexpressed. Figure S8. Survival curves obtained for immune groups. Figure S9. Immune node activities in immune groups. Figure S10. Concordance between classification of layer 1, which divided tumors into Luminal and Basal, and layer 3, which divided tumors into immune-high and immune-low group. Figure S11. Heatmap showing differences between groups defined in layers 14 and 15. Green = underexpressed, Red = overexpressed. Figure S12. Different classifications obtained by each layer. Colour bars represent the assignation for each patient and the numbers on the righ are the corresponding layers. For instance, layer 12 classified the patients into two groups (light blue and dark blue). Figure S13. Flux activities of luminal and basal groups. Table S1. Clinical patients’ characteristics. Table S3. Main gene ontology term defined for each sixteen layers obtained by the sparse k-means-CCA workflow. Table S4. Percentages of patients assigned to each group in TCGA and layer classification. Table S5. Number and percentage of tumors assigned to each group by the layer classification. EM = extracellular matrix, IM = immune. (DOCX 2320 kb) [file 12885_2019_5858_MOESM1_ESM.docx]

**Supplementary information**

S1 Fig: Flowchart for patient selection.


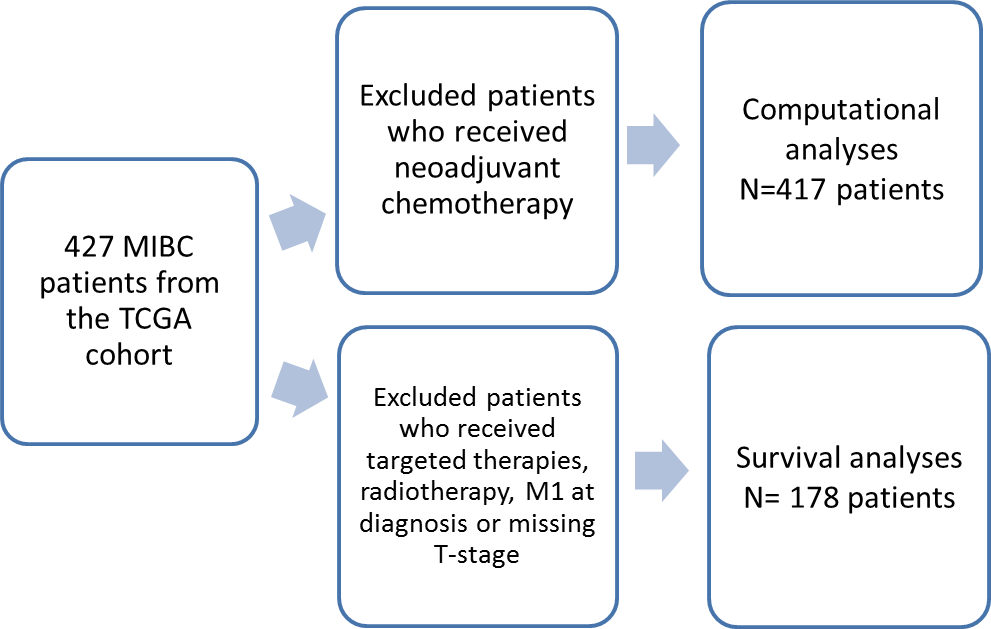


S2 Fig: PGM’s graph heatmap showing differences between Group 1.1 (Luminal) and Group 1.2 (Basal). Green= underexpressed, Red= overexpressed.


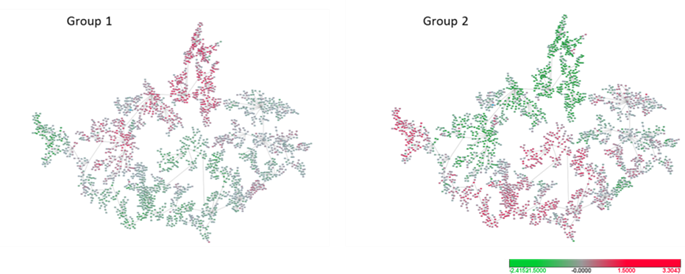


S3 Fig: Kaplan Meier analysis comparing Luminal and Basal MIBC tumors clinical evolution.





S4 Fig: Functional node activities comparison between Luminal and Basal group.





S5 Fig: PGM’s graph heatmap showing differences between Group 2.1 and Group 2.2. Green= underexpressed, Red= overexpressed.


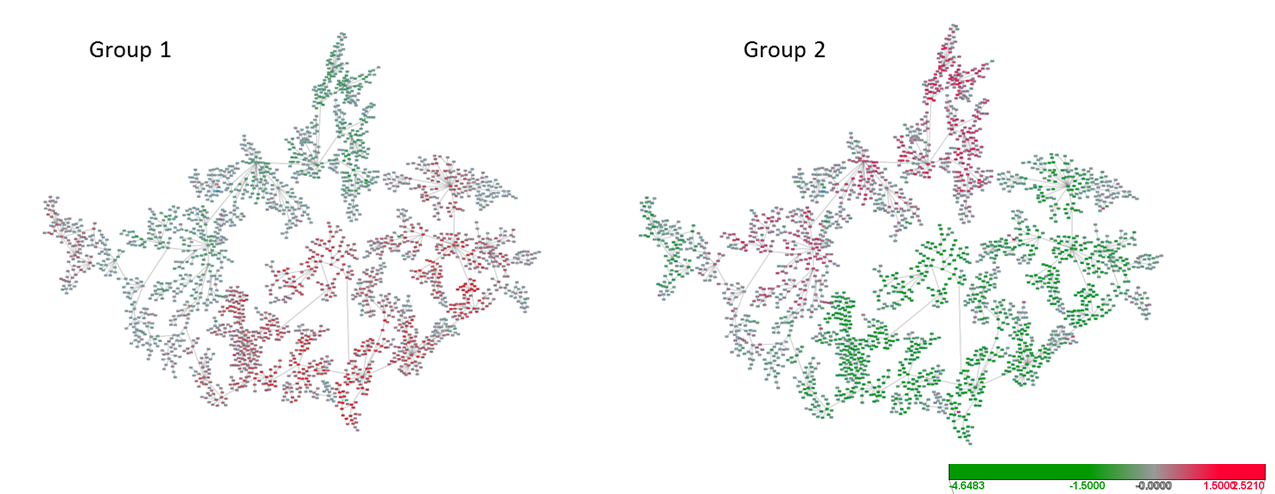


S6 Fig: Kaplan Meier analysis comparing Group 2.1 and Group 2.2 clinical evolution.





S7 Fig: Heatmap showing differences between Group 3.1 (immune-high) and Group 3.2 (immune-low). Green= underexpressed, Red= overexpressed.


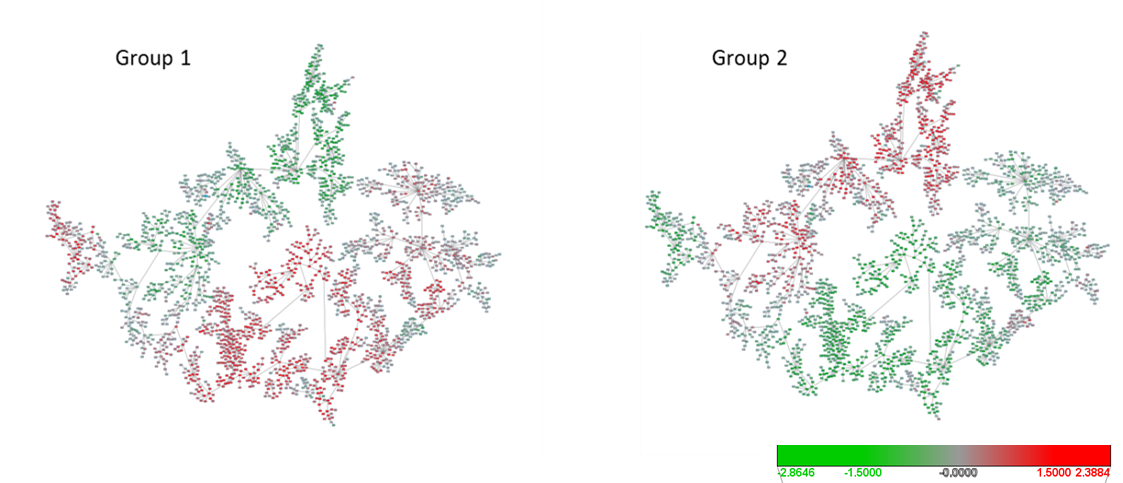


S8 Fig: Survival curves obtained for immune groups.





S9 Fig: Immune node activities in immune groups.





S10 Fig: Concordance between classification of layer 1, which divided tumors into Luminal and Basal, and layer 3, which divided tumors into immune-high and immune-low group.

S11 Fig: Heatmap showing differences between groups defined in layers 14 and 15. Green= underexpressed, Red= overexpressed.


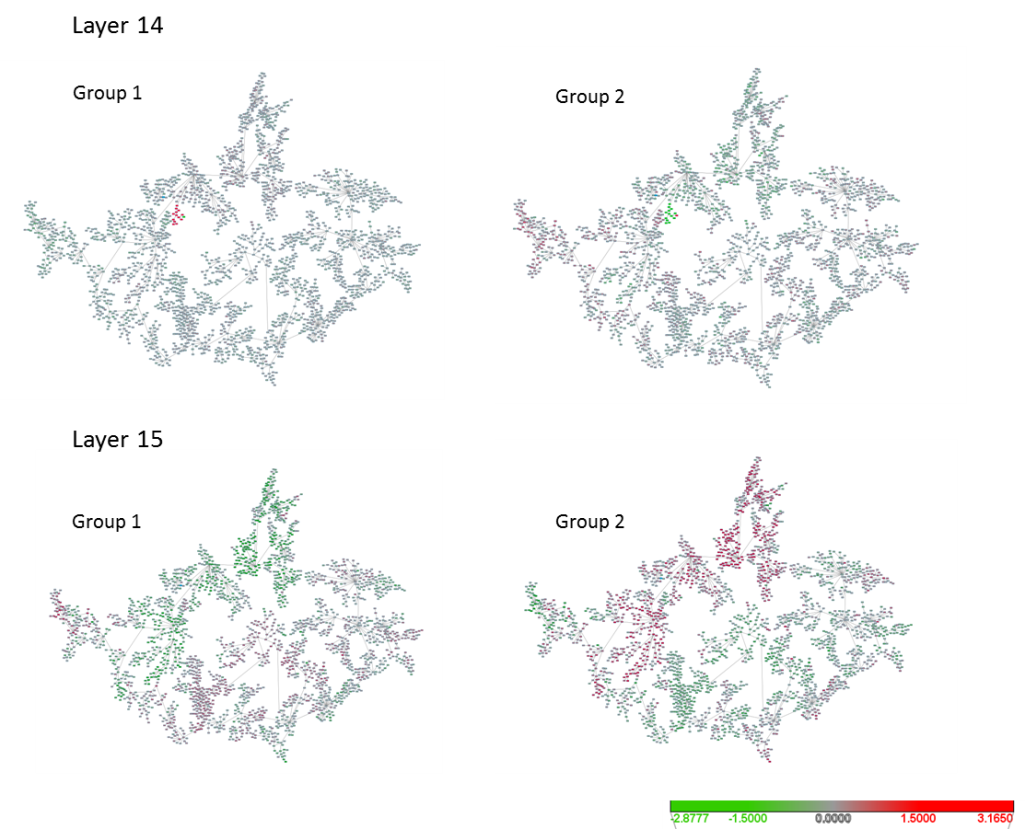


S12 Fig: Different classifications obtained by each layer. Colour bars represent the assignation for each patient and the numbers on the righ are the corresponding layers. For instance, layer 12 classified the patients into two groups (light blue and dark blue).


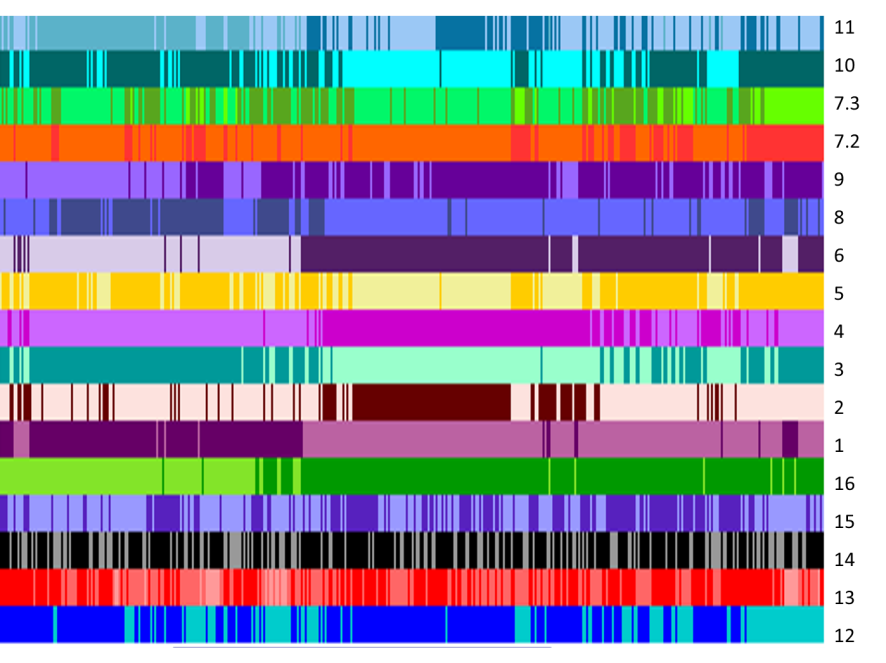


S13 Fig: Flux activities of luminal and basal groups.





S1 Table: Clinical patients’ characteristics.

| Muscle-invasive bladder tumors | n |  | Percentages (%) |
| --- | --- | --- | --- |
| Number of patients | 178 |  |  |
| Age (years) |  |  |  |
| ≤60 | 43 |  | 24.2 |
| >60 | 135 |  | 75.8 |
| Median | 68.5 |  |  |
| Range | 37-90 |  |  |
| Sex |  |  |  |
| Male | 131 |  | 73.6 |
| Female | 47 |  | 26.4 |
| pT category |  |  |  |
| pT2unknown | 9 |  | 5.1 |
| pT2a | 18 |  | 10.1 |
| pT2b | 31 |  | 17.4 |
| pT3unknown | 23 |  | 12.9 |
| pT3a | 21 |  | 11.8 |
| pT3b | 38 |  | 21.3 |
| pT4unknown | 8 |  | 4.5 |
| pT4a | 17 |  | 9.6 |
| Missing | 13 |  | 7.3 |
| pN category |  |  |  |
| pN0 | 110 |  | 61.8 |
| pN1 | 22 |  | 12.4 |
| pN2 | 31 |  | 17.4 |
| pN3 | 5 |  | 2.8 |
| Missing | 10 |  | 5.6 |
| Grade |  |  |  |
| Low grade | 6 |  | 3.4 |
| High grade | 170 |  | 95.5 |
| Missing | 2 |  | 1.1 |

S3 Table: Main gene ontology term defined for each sixteen layers obtained by the sparse k-means-CCA workflow.

| Biological layer | Number of genes | Gene Ontology |
| --- | --- | --- |
| 1 | 75 | Extracellular exosome, epidermis development, sodium ion homeostasis |
| 2 | 82 | Extracellular space |
| 3 | 66 | Inflammatory response |
| 4 | 72 | Innate immune response |
| 5 | 129 | Extracellular region |
| 6 | 122 | Epidermis development |
| 7 | 106 | Extracellular matrix |
| 8 | 83 | Keratinocyte differentiation, Metabolism of xenobiotics by cytochrome P450 |
| 9 | 76 | Epidermis development, extracellular matrix |
| 10 | 148 | Extracellular region, T cell receptor complex |
| 11 | 121 | Extracellular region |
| 12 | 146 | Cell adhesion, plasma membrane |
| 13 | 61 | Extracellular exosome |
| 14 | 14 | Translational initiation |
| 15 | 10 | Chemical synaptic transmission |
| 16 | 133 | Plasma membrane |

S4 Table: Percentages of patients assigned to each group in TCGA and layer classification.

|  | %TCGA classification | %layer classification |
| --- | --- | --- |
| Luminal | 60.00% | 62.35% |
| Basal | 35.00% | 37.64% |
| Immune positive | 54.00% | 51.55% |
| Immune negative | 46.00% | 48.44% |

S5 Table: Number and percentage of tumors assigned to each group by the layer classification. EM = extracellular matrix, IM= immune

| **Layer 1** | **Layer 2** | **Layer 3** |
| --- | --- | --- |
| Luminal  n= 260 (62%) | EM-low  n= 122 (29%) | IM-low  n= 118 (28%) |
|  |  | IM-high  n= 4 (1%) |
|  | EM-high  n= 138 (33%) | IM-low  n = 69 (17%) |
|  |  | IM-high  n = 69 (17%) |
| Basal  n= 157 (37%) | EM-low  n= 27 (7%) | IM-low  n = 10 (2%) |
|  |  | IM-high  n = 17 (4%) |
|  | EM-high  n= 130 (31%) | IM-low  n = 5 (1%) |
|  |  | IM-high  n =125 (30%) |
